# 1916.—A Year's Work in the Hospitals and Medical Charities of London

**Published:** 1917-06-23

**Authors:** 


### 1916.

### A YEAR'S WORK IN THE HOSPITALS AND MEDICAL CHARITIES OF LONDON.

### ST. MARYLEBONE AND WEST CENTRAL DISTRICT.

Comprising St. Marylebone, St. John's Wood, Bloomsbury, Holborn, &c.

| No. of Beds. | No. of Beds Daily Occupied. | HOSPITALS. | In-patients. | Out-patient Attendances. | Total Expenditure. | Income. | | | Total Income. | Legacies not included in preceding column. |
|---|---|---|---|---|---|---|---|---|---|---|
| | | | | | | Charitable. | Proprietary. | Patients' Payments. | | |
| | | | | | £ | £ | £ | £ | £ | £ |
| 74 | 55 | French ... ... ... ... | 802 | 20,152 | 6,949 | 5,719 | 1,854 | 1,132 | 8,705 | 1,316 |
| 85 | 65 | Italian ... ... ... ... | 903 | 7,363 | 6,296 | 2,252 | 2,257 | 3,048 | 7,557 | 355 |
| 165 | 130 | London Homœopathic ... ... | 1,548 | 55,054 | 17,357 | 4,679 | 5,135 | 4,952 | 14,766 | 2,296 |
| 151 | 135 | SS. John and Elizabeth ... ... | 1,184 | ... | 15,947 | 1,827 | 7,927 | 4,896 | 14,650 | 120 |
| 474 | 358 | The Middlesex ... ... ... | 7,319 | 156,578 | 52,642 | 20,144 | 12,296 | 4,940 | 37,380 | 11,420 |
| 90 | 89 | Alexandra, for Children... ... | 95 | 1,003 | 5,740 | 3,182 | 1,352 | 3,100 | 7,634 | 200 |
| 240 | 232 | Hospital for Sick Children ... | 2,810 | 91,441 | 27,890 | 12,248 | 6,889 | 2,060 | 21,197 | 2,476 |
| 24 | 20 | S. Monica's, for Children ... | 126 | ... | 1,514 | 886 | 169 | 291 | 1,346 | 250 |
| 82 | 77 | Queen Charlotte's Lying-In ... | 2,206 | 20,580 | 9,189 | 7,444 | 1,256 | 1,201 | 9,901 | 83 |
| 71 | 66 | New Hospital for Women ... | 1,281 | 34,114 | 9,329 | 2,914 | 1,236 | 2,704 | 6,854 | 535 |
| 70 | 52 | Samaritan Free ... ... ... | 1,035 | 12,195 | 7,103 | 8,552 | 604 | ... | 9,156 | 881 |
| 207 | 191 | National for the Paralysed, &c.... | 1,026 | 47,170 | 27,003 | 10,490 | 2,786 | 10,600 | 23,876 | 2,180 |
| 85 | 81 | Hospital for Epilepsy, &c. ... | 493 | 21,751 | 8,385 | 5,230 | 247 | 3,536 | 9,013 | 1,025 |
| 82 | 78 | West End, for Epilepsy, &c. ... | 318 | 42,021 | 10,016 | 4,670 | 1,781 | 3,276 | 9,727 | 15 |
| 40 | 22 | Central London Ophthalmic ... | 412 | 28,097 | 3,302 | 2,883 | 71 | ... | 2,954 | 600 |
| 16 | 14 | Western Ophthalmic ... ... | 408 | 25,600 | 2,255 | 1,663 | 139 | 430 | 2,232 | ... |
| 200 | 164 | Royal National Orthopædic ... | 1,373 | 28,009 | 17,060 | 6,693 | 1,525 | 9,330 | 17,548 | 134 |
| 38 | 32 | Florence Nightingale Home ... | 486 | ... | 4,960 | 1,562 | 307 | 2,989 | 4,858 | ... |
| 14 | 6 | London Throat ... ... ... | 289 | 9,143 | 1,602 | 439 | 111 | 741 | 1,291 | ... |
| 49 | 47 | The Middlesex Cancer ... ... | 171 | 439 | 6,383 | 3,367 | 2,798 | ... | 6,165 | 644 |
| 20 | 13 | Metropolitan Ear, &c. ... ... | 364 | 12,553 | 2,236 | 833 | 91 | 1,391 | 2,315 | ... |
| 2,277 | 1,927 | | 24,649 | 613,263 | 243,158 | 107,677 | 50,831 | 60,617 | 219,125 | 24,530 |
| | | DISPENSARIES. | | | | | | | | |
| ... | ... | London Medical Mission ... | ... | 23,771 | 1,691 | 556 | 389 | 333 | 1,278 | 11 |
| ... | ... | Margaret Street, for Consumption | ... | 7,967 | 1,090 | 453 | 401 | ... | 854 | ... |
| ... | ... | St. John's Wood Provident ... | ... | 15,093 | 460 | 244 | 20 | 162 | 426 | ... |
| ... | ... | St. Marylebone General ... ... | ... | 14,939 | 1,435 | 574 | 242 | 318 | 1,134 | ... |
| ... | ... | Western General ... ... ... | ... | 13,132 | 924 | 578 | 146 | 17 | 741 | ... |
| 2,277 | 1,927 | | 24,649 | 688,165 | 248,758 | 110,082 | 52,029 | 61,447 | 223,558 | 24,541 |

### WESTMINSTER DISTRICT.—Comprising Westminster City and Liberties.

| | | | | | | | | | | |
|---|---|---|---|---|---|---|---|---|---|---|
| | | HOSPITALS. | | | £ | £ | £ | £ | £ | £ |
| 300 | 234 | Charing Cross ... ... ... | 3,174 | 81,132 | 40,336 | 30,652 | 4,821 | 7,995 | 43,468 | 335 |
| 213 | 191 | Westminster ... ... ... | 1,949 | 59,277 | 28,899 | 8,847 | 5,308 | 5,863 | 20,018 | 7,455 |
| 160 | 147 | Ventnor, for Consumption ... | 493 | ... | 16,981 | 7,350 | 3,535 | 7,046 | 17,931 | 124 |
| 37 | 30 | Grosvenor, for Women & Children | 464 | 5,801 | 3,457 | 1,640 | 409 | 1,074 | 3,123 | ... |
| 67 | 65 | Hospital for Women ... ... | 1,035 | 12,638 | 7,231 | 4,523 | 600 | 1,518 | 6,641 | 735 |
| 25 | 18 | Gordon, for Fistula ... ... | 312 | 3,199 | 2,366 | 550 | 51 | 1,766 | 2,367 | ... |
| 24 | 24 | National, for Diseases of Heart... | 164 | 17,079 | 4,620 | 2,205 | 819 | 940 | 3,964 | 59 |
| 40 | 31 | Royal Westminster Ophthalmic... | 751 | 38,532 | 4,229 | 2,303 | 1,352 | 123 | 3,778 | 455 |
| ... | ... | Royal Dental ... ... ... | ... | 37,239 | 5,304 | 4,223 | 1,016 | 1,239 | 6,478 | 20 |
| 32 | 28 | St. Peter's, for Stone ... ... | 452 | 35,561 | 5,127 | 1,786 | 696 | 2,625 | 5,107 | 5 |
| 50 | 33 | Infants' Hospital, Vincent Square | 357 | 3,735 | 3,728 | 2,914 | 73 | ... | 2,987 | ... |
| 40 | 28 | St. John's, for Skin Diseases ... | 180 | 37,076 | 4,455 | 3,479 | 51 | 2,038 | 5,568 | 50 |
| 57 | 32 | Throat Hospital, Golden Square | 794 | 50,856 | 6,476 | 2,747 | 62 | 4,026 | 6,835 | 80 |
| 1,045 | 861 | | 10,125 | 382,125 | 133,209 | 73,219 | 18,793 | 36,253 | 128,265 | 9,318 |
| | | DISPENSARIES. | | | | | | | | |
| ... | ... | Public ... ... ... ... | ... | 10,321 | 728 | 340 | 207 | ... | 547 | 50 |
| ... | ... | St. George's, Hanover Square ... | ... | 1,849 | 414 | 249 | 84 | 72 | 405 | ... |
| ... | ... | Western ... ... ... ... | ... | 16,068 | 1,341 | 266 | 502 | 607 | 1,375 | ... |
| ... | ... | Westminster General ... ... | ... | 14,932 | 797 | 305 | 347 | 100 | 752 | ... |
| 1,045 | 861 | | 10,125 | 425,295 | 136,489 | 74,379 | 19,933 | 37,032 | 131,344 | 9,368 |

### CITY AND EAST CENTRAL DISTRICT.

Comprising the City, St. Luke's, Shoreditch, Finsbury, and Clerkenwell.

| No. of Beds. | No. of Beds Daily Occupied. | HOSPITALS. | In-patients. | Out-patient Attendances. | Total Expenditure. | Income. Charitable. | Income. Proprietary. | Income. Patients' Payments. | Total Income. | Legacies not included in preceding column. |
|---|---|---|---|---|---|---|---|---|---|---|
| | | | | | £ | £ | £ | £ | £ | £ |
| 373 | 312 | Metropolitan ... ... ... | 3,769 | 90,572 | 32,806 | 14,308 | 1,483 | 16,277 | 32,068 | 2,042 |
| 175 | 152 | Royal Free ... ... ... | 2,339 | 115,913 | 35,607 | 8,847 | 4,276 | 2,592 | 15,715 | 35,618 |
| 689 | 560 | St. Bartholomew's ... ... | 8,007 | 261,298 | 101,332 | 11,729 | 78,124 | 12,072 | 101,925 | 1,507 |
| 81 | 47 | Royal, for Diseases of the Chest. | 503 | 15,987 | 9,006 | 6,050 | 436 | 3,467 | 9,953 | 126 |
| 170 | 170 | Queen's, for Children ... ... | 2,161 | 108,432 | 20,154 | 17,497 | 2,428 | 635 | 20,560 | 3,070 |
| 61 | 51 | City of London Lying-In ... | 1,251 | 7,254 | 7,613 | 3,063 | 2,864 | 962 | 6,889 | 50 |
| 52 | 44 | St. Mark's, for Fistula ... ... | 714 | 5,524 | 6,184 | 3,984 | 1,361 | 6 | 5,351 | 191 |
| 138 | 112 | Royal London Ophthalmic ... | 2,396 | 124,095 | 16,520 | 10,502 | 1,959 | 1,710 | 14,171 | 4,128 |
| 42 | 31 | Central London Throat and Ear. | 573 | 42,998 | 5,207 | 1,371 | 284 | 3,966 | 5,621 | 20 |
| 1,781 | 1,479 | | 21,713 | 772,073 | 234,429 | 77,351 | 93,215 | 41,687 | 212,253 | 46,752 |
| | | DISPENSARIES. | | | | | | | | |
| ... | ... | Billingsgate Medical Mission ... | ... | 9,477 | 656 | 437 | 30 | 72 | 539 | 50 |
| ... | ... | City ... ... ... ... | ... | 16,290 | 906 | 681 | 103 | ... | 784 | 100 |
| ... | ... | Farringdon General ... ... | ... | 7,227 | 506 | 340 | 23 | 190 | 553 | ... |
| ... | ... | Finsbury ... ... ... ... | ... | 26,510 | 1,057 | 473 | 480 | 201 | 1,154 | ... |
| ... | ... | Metropolitan ... ... ... | ... | 2,774 | 628 | 268 | 255 | 82 | 605 | ... |
| ... | ... | Royal General ... ... ... | ... | 7,980 | 937 | 791 | 390 | 57 | 1,238 | ... |
| 1,781 | 1,479 | | 21,713 | 842,331 | 239,119 | 80,341 | 94,496 | 42,289 | 217,126 | 46,290 |

### ISLINGTON AND NORTH-WEST DISTRICT.

Comprising Islington, Holloway, Highbury, Hampstead, Highgate, St. Pancras, Stoke Newington, Tottenham, &c.

| No. of Beds. | No. of Beds Daily Occupied. | HOSPITALS. | In-patients. | Out-patient Attendances. | Total Expenditure. | Income. Charitable. | Income. Proprietary. | Income. Patients' Payments. | Total Income. | Legacies not included in preceding column. |
|---|---|---|---|---|---|---|---|---|---|---|
| | | | | | £ | £ | £ | £ | £ | £ |
| 275 | 244 | Great Northern Central ... ... | 3,021 | 102,525 | 34,591 | 19,066 | 1,907 | 11,517 | 32,490 | 693 |
| 137 | 106 | Hampstead General Hospital ... | 1,246 | 31,424 | 15,293 | 7,322 | 3,310 | 3,898 | 14,530 | 518 |
| 116 | 95 | London Temperance ... ... | 1,156 | 81,537 | 11,133 | 5,670 | 1,967 | 1,864 | 9,501 | 150 |
| 125 | 100 | Tottenham (Prince of Wales's)... | 1,712 | 82,430 | 14,393 | 12,073 | 915 | 473 | 13,461 | 2,105 |
| 464 | 414 | University College ... ... | 5,090 | 159,639 | 41,655 | 18,379 | 6,121 | 15,481 | 39,981 | 3,478 |
| 110 | 108 | Mount Vernon, for Consumption | 357 | 13,354 | 12,995 | 2,348 | 3,538 | 5,858 | 11,744 | 25 |
| 20 | 18 | Children's Home Hospital, Barnet | 52 | ... | 639 | 429 | 234 | 82 | 745 | ... |
| 160 | 54 | London Fever ... ... ... | 897 | ... | 12,493 | 8,025 | 1,505 | 3,944 | 13,474 | 1,939 |
| 22 | 5 | Bushey Heath Cottage ... ... | 88 | ... | 689 | 301 | 95 | 110 | 506 | 250 |
| 25 | 21 | Enfield Cottage ... ... ... | 232 | ... | 1,542 | 948 | 205 | 225 | 1,378 | ... |
| 25 | 15 | St. Saviour's Hospital ... ... | 182 | ... | 2,236 | 1,581 | 197 | 1,095 | 2,873 | ... |
| 48 | 39 | St. Columba's Hospital ... ... | 159 | ... | 4,417 | 2,422 | 353 | 470 | 3,245 | 105 |
| 62 | 46 | Willesden Cottage ... ... | 488 | 1,855 | 3,578 | 1,460 | 207 | 2,170 | 3,837 | ... |
| 30 | 18 | Wood Green Cottage ... ... | 264 | 58 | 1,736 | 1,114 | 80 | 560 | 1,754 | 100 |
| 20 | 20 | Santa Claus Home ... ... | 21 | ... | 1,028 | 932 | 92 | 63 | 1,087 | ... |
| 26 | 16 | Stoke Newington Home Hospital | 183 | ... | 1,484 | 575 | 487 | 307 | 1,369 | ... |
| 56 | 54 | Hospital for Incurable Children | 25 | ... | 2,385 | 634 | 634 | 1,115 | 2,383 | 100 |
| 18 | 17 | Winifred House, Holloway ... | 21 | ... | 874 | 460 | 56 | 201 | 717 | ... |
| 18 | 17 | Highgate Convalescent Home ... | 138 | ... | 499 | 502 | 19 | 4 | 525 | ... |
| 76 | 62 | St. Andrew's, Dollis Hill ... | 343 | ... | 7,224 | 1,429 | 964 | 4,742 | 7,135 | 20 |
| 1,833 | 1,469 | | 15,675 | 472,822 | 170,884 | 85,670 | 22,886 | 54,179 | 162,735 | 9,483 |
| | | DISPENSARIES | | | | | | | | |
| ... | ... | Child's Hill Provident ... ... | ... | 3,100 | 87 | 15 | 9 | 62 | 86 | ... |
| ... | ... | Hampstead Provident ... ... | ... | 8,752 | 763 | 236 | 55 | 507 | 798 | ... |
| ... | ... | Islington ... ... ... ... | ... | 44,470 | 806 | 146 | 90 | 555 | 791 | 100 |
| ... | ... | Islington Medical Mission ... | ... | 11,168 | 842 | 568 | 308 | 94 | 970 | ... |
| ... | ... | St. Pancras and Northern ... | ... | 10,139 | 1,265 | 371 | 126 | 987 | 1,484 | 280 |
| ... | ... | Stamford Hill, &c. ... ... | ... | 19,831 | 591 | 453 | 154 | ... | 607 | ... |
| 1,833 | 1,469 | | 15,675 | 570,282 | 175,238 | 87,459 | 23,628 | 56,384 | 167,471 | 9,863 |

### STRATFORD AND EAST-END DISTRICT.

**Comprising Bethnal Green, Tower Hamlets, West Ham, Whitechapel, Hackney, Stepney, Limehouse, Poplar, and the East.**

| No. of Beds. | No. of Beds Daily Occupied. | Hospitals. | In-patients | Out-patient Attendances. | Total Expenditure. | Income. Charitable. | Income. Proprietary. | Income. Patients' Payments. | Total Income. | Legacies not included in preceding column. |
|---|---|---|---|---|---|---|---|---|---|---|
| | | | | | £ | £ | £ | £ | £ | £ |
| 184 | 153 | German ... ... ... ... | 1,509 | 33,613 | 15,366 | 5,189 | 5,126 | 1,346 | 11,661 | 2,320 |
| 1,100 | 813 | London ... ... ... ... | 16,791 | 478,166 | 183,718 | 85,664 | 40,158 | 11,454 | 137,276 | 18,196 |
| 50 | 20 | Mildmay Mission Hospital ... | 320 | 20,903 | 3,870 | 5,467 | 599 | 142 | 6,208 | ... |
| 30 | 21 | Mildmay Memorial ... ... | 267 | ... | 2,242 | 1,055 | 977 | 239 | 2,271 | ... |
| 115 | 85 | Poplar ... ... ... ... | 1,802 | 67,639 | 12,478 | 12,370 | 3,030 | 1,604 | 17,004 | 1,071 |
| 46 | 37 | Walthamstow, &c. ... ... | 465 | 10,997 | 2,638 | 2,183 | 510 | 32 | 2,725 | 12 |
| 148 | 137 | Queen Mary's, &c. ... ... | 1,810 | 127,885 | 17,528 | 15,307 | 1,344 | 2,637 | 19,288 | 1,896 |
| 175 | 153 | City of London for Dis. of the Chest | 782 | 31,249 | 17,216 | 10,614 | 1,103 | 2,026 | 13,743 | 3,262 |
| 126 | 116 | East London, for Children ... | 1,600 | 63,057 | 12,347 | 15,930 | 1,722 | ... | 17,652 | 1,009 |
| 43 | 38 | St. Mary's, Plaistow, for Children | 535 | 19,008 | 3,864 | 3,557 | 517 | 65 | 4,139 | 554 |
| 33 | 25 | East End Mothers' Home ... | 679 | 22,650 | 3,210 | 1,639 | 1,217 | 278 | 3,134 | ... |
| 20 | 17 | Plaistow Maternity ... ... | 447 | ... | 1,084 | 485 | 9 | 303 | 797 | 1 |
| 26 | 19 | Canning Town Cottage ... ... | 200 | 11,496 | 2,407 | 1,725 | 301 | 428 | 2,454 | 33 |
| 17 | 10 | Passmore Edwards Cottage, T'lb'ry | 183 | 1,546 | 1,039 | 1,106 | 95 | 21 | 1,222 | ... |
| 25 | 13 | East Ham Cottage ... ... | 127 | 13,869 | 1,310 | 522 | 970 | 67 | 1,559 | ... |
| 2,048 | 1,657 | | 27,517 | 902,078 | 280,317 | 162,813 | 57,678 | 20,642 | 241,133 | 28,354 |
| | | **Dispensaries.** | | | | | | | | |
| ... | ... | All Saints', Buxton Street ... | ... | 1,858 | 98 | 94 | ... | ... | 94 | ... |
| ... | ... | Eastern ... ... ... ... | ... | 23,803 | 847 | 119 | 287 | 394 | 800 | ... |
| ... | ... | London ... ... ... ... | ... | 1,889 | 417 | 73 | 244 | ... | 317 | ... |
| ... | ... | Mildmay Medical Mission ... | ... | 2,723 | 258 | 152 | ... | 51 | 203 | ... |
| ... | ... | Queen Adelaide's ... ... ... | ... | 10,888 | 531 | 366 | 405 | ... | 771 | ... |
| 2,048 | 1,657 | | 27,517 | 943,239 | 282,468 | 163,617 | 58,614 | 21,087 | 243,318 | 28,354 |

### KENSINGTON AND WEST DISTRICT.

**Comprising Kensington, Paddington, Bayswater, Kilburn, Chelsea, Brompton, Fulham, Hammersmith, Chiswick, Brentford, Acton, Ealing, &c.**

| | | | | | | | | | | |
|---|---|---|---|---|---|---|---|---|---|---|
| | | **Hospitals.** | | | £ | £ | £ | £ | £ | £ |
| 349 | 311 | St. George's ... ... ... | 4,359 | 108,817 | 49,127 | 14,499 | 12,195 | 3,996 | 30,690 | 34,725 |
| 298 | 247 | St. Mary's ... ... ... ... | 3,288 | 90,233 | 37,441 | 13,008 | 10,897 | 2,921 | 26,826 | 27,153 |
| 172 | 170 | West London ... ... ... | 2,269 | 141,403 | 21,465 | 14,024 | 1,462 | 2,453 | 17,939 | 2,405 |
| 483 | 453 | Hospital for Consumption ... | 1,664 | 29,374 | 49,412 | 22,034 | 3,805 | 14,240 | 40,079 | 6,694 |
| 40 | 38 | Belgrave, for Children ... ... | 686 | 37,968 | 4,954 | 4,254 | 620 | 99 | 4,973 | ... |
| 82 | 79 | Cheyne, for Sick & Incurable Chldn | 59 | ... | 4,745 | 1,661 | 1,460 | 591 | 3,712 | 104 |
| 24 | 20 | Kensington, General ... ... | 319 | 27,215 | 3,142 | 1,963 | 339 | 899 | 3,201 | 15 |
| 13 | 9 | Kensington, for Children ... | 152 | 9,267 | 1,281 | 836 | 220 | ... | 1,056 | 200 |
| 46 | 35 | Paddington Green, for Children | 735 | 47,093 | 6,772 | 4,540 | 5,022 | 381 | 9,943 | 250 |
| 169 | 143 | Victoria, for Children ... ... | 1,294 | 61,223 | 12,289 | 7,813 | 2,466 | 595 | 10,874 | 918 |
| 57 | 44 | Chelsea, for Women ... ... | 800 | 8,971 | 8,997 | 2,903 | 848 | 1,235 | 4,986 | 975 |
| 127 | 99 | Cancer ... ... ... ... | 798 | 1,519 | 21,352 | 3,128 | 9,059 | ... | 12,187 | 23,103 |
| 145 | 126 | Female Lock ... ... ... | 894 | ... | 8,168 | 6,856 | 190 | 1,968 | 9,014 | 7,894 |
| 24 | 22 | Banstead Surgical Home ... | 107 | ... | 951 | 421 | 48 | 543 | 1,012 | ... |
| 30 | 30 | Acton Cottage ... ... ... | 341 | 8,626 | 2,089 | 1,992 | 237 | 591 | 2,820 | ... |
| 70 | 35 | Ealing Cottage ... ... ... | 531 | ... | 3,623 | 2,525 | 431 | 1,096 | 4,052 | 25 |
| 16 | 10 | Epsom and Ewell Cottage ... | 176 | ... | 898 | 574 | 61 | 201 | 836 | ... |
| 27 | 21 | Hounslow Cottage ... ... | 296 | 3,990 | 1,535 | 611 | 311 | 484 | 1,406 | ... |
| 40 | 37 | Reigate and Redhill Cottage ... | 637 | ... | 3,124 | 2,358 | 263 | 430 | 3,051 | ... |
| 24 | 19 | Teddington Cottage ... ... | 298 | ... | 1,266 | 1,016 | 244 | 100 | 1,360 | ... |
| 26 | 22 | Wimbledon Cottage ... ... | 376 | ... | 2,241 | 1,444 | 187 | 933 | 2,564 | 1,000 |
| 35 | 30 | Wimbledon, Nelson Hospital ... | 552 | 1,560 | 2,184 | 1,332 | 211 | 682 | 2,225 | 1,000 |
| 2.297 | 2,000 | | 20,631 | 577,259 | 247,056 | 109,792 | 50,576 | 34,438 | 194,806 | 106,461 |
| | | **Dispensaries.** | | | | | | | | |
| ... | ... | Kilburn, Maida Vale ... ... | ... | 4,575 | 364 | 328 | 29 | ... | 357 | ... |
| ... | ... | Kilburn Provident ... ... | ... | 11,972 | 1,151 | 99 | 19 | 1,043 | 1,161 | ... |
| ... | ... | Notting Hill Provident ... ... | ... | 5,997 | 469 | 82 | 26 | 381 | 489 | ... |
| ... | ... | Paddington Provident ... ... | ... | 2,585 | 240 | 98 | 1 | 141 | 240 | ... |
| ... | ... | Royal Pimlico Provident... ... | ... | 6,936 | 426 | 210 | 93 | 57 | 360 | ... |
| ... | ... | Westbourne Provident ... ... | ... | 1,965 | 193 | 49 | 5 | 158 | 212 | ... |
| 2,297 | 2,000 | | 20,631 | 611,289 | 249,899 | 110,658 | 50,749 | 36,218 | 197,625 | 106,461 |

### NEWINGTON AND SOUTH DISTRICT.

**Comprising Battersea, Wandsworth, Tooting, Balham, Streatham, Brixton, Lambeth, Newington, Southwark, Bermondsey, Camberwell, Greenwich, Deptford, Lewisham, Blackheath, Woolwich, &c.**

| No. of Beds. | No. of Beds Daily Occupied. | Hospitals. | In-patients. | Out-patient Attendances. | Total Expenditure. | Income. Charitable. | Income. Proprietary. | Income. Patients' Payments. | Total Income. | Legacies not included in preceding column. |
|---|---|---|---|---|---|---|---|---|---|---|
| | | | | | £ | £ | £ | £ | £ | £ |
| 643 | 559 | Guy's ... ... ... ... | 8,806 | 428,944 | 96,326 | 20,590 | 49,861 | 5,436 | 75,887 | 27,582 |
| 687 | 544 | King's College ... ... ... | 8,146 | 104,860 | 76,844 | 13,435 | 33,026 | 27,254 | 73,715 | 1,253 |
| 18 | 11 | Phillips' Memorial Homœopathic | 146 | 2,111 | 1,352 | 489 | 395 | 412 | 1,296 | ... |
| 66 | 70 | Miller ... ... ... ... | 1,034 | 88,361 | 9,886 | 8,931 | 855 | 558 | 10,344 | 100 |
| 60 | 47 | St. John's, Lewisham ... ... | 444 | 11,151 | 4,512 | 1,886 | 577 | 1,542 | 4,005 | 141 |
| 1,014 | 763 | St. Thomas's ... ... ... | 9,659 | 234,812 | 107,942 | 5,506 | 67,496 | 30,049 | 103,051 | 847 |
| 395 | 317 | Seamen's ... ... ... ... | 3,115 | 50,770 | 30,403 | 21,274 | 5,751 | 11,581 | 38,606 | 1,175 |
| 60 | 50 | Bolingbroke Hospital ... ... | 650 | 26,088 | 7,734 | 3,051 | 1,391 | 1,040 | 5,482 | 100 |
| 76 | 49 | Evelina, for Children ... ... | 917 | 40,611 | 8,996 | 2,425 | 4,508 | 242 | 7,175 | 1,225 |
| 57 | 47 | Home for Sick Children... ... | 316 | 6,275 | 3,140 | 1,332 | 284 | 607 | 2,223 | 50 |
| 14 | 10 | British Home for Mothers and Babies ... ... ... ... | 194 | 2,546 | 1,399 | 912 | 398 | 509 | 1,819 | 25 |
| 36 | 33 | General Lying-In... ... ... | 876 | 7,766 | 6,042 | 3,379 | 2,987 | ... | 6,366 | ... |
| 50 | 37 | Clapham Maternity & Dispensary | 904 | 4,656 | 3,094 | 878 | 883 | 1,727 | 3,488 | 50 |
| 66 | 45 | South London, for Women ... | 227 | 24,920 | 4,159 | 6,258 | 1,066 | 768 | 8,092 | ... |
| 116 | 87 | Royal Waterloo ... ... ... | 1,139 | 17,962 | 8,630 | 6,108 | 1,928 | 3 | 8,039 | 165 |
| 42 | 36 | Royal Eye ... ... ... ... | 593 | 59,875 | 5,756 | 2,145 | 1,145 | 1,647 | 4,937 | 138 |
| 28 | 16 | Beckenham Cottage ... ... | 304 | 485 | 1,653 | 1,170 | 18 | 328 | 1,516 | 310 |
| 26 | 21 | Blackheath Cottage ... ... | 252 | 3,059 | 2,060 | 1,284 | 275 | 577 | 2,136 | ... |
| 42 | 18 | Bromley Cottage ... ... ... | 324 | 273 | 2,257 | 1,075 | 494 | 486 | 2,055 | ... |
| 27 | 17 | Chislehurst, &c., Cottage ... | 263 | ... | 1,823 | 1,489 | 175 | 522 | 2,186 | ... |
| 22 | 11 | Eltham Cottage ... ... ... | 195 | ... | 1,088 | 738 | 132 | 277 | 1,147 | 50 |
| 12 | 7 | Sidcup Cottage ... ... ... | 101 | 80 | 670 | 361 | 68 | 183 | 612 | ... |
| 34 | 24 | Livingstone Cottage ... ... | 336 | ... | 1,657 | 1,501 | 131 | 84 | 1,716 | ... |
| 23 | 17 | Victoria Hospital, Kingston ... | 308 | ... | 1,438 | 927 | 238 | 285 | 1,450 | 425 |
| 3,614 | 2,836 | | 39,249 | 1,115,605 | 388,861 | 107,144 | 174,082 | 86,117 | 367,343 | 33,636 |
| | | **DISPENSARIES.** | | | | | | | | |
| ... | ... | Battersea Provident ... ... | ... | 111,000 | 3,568 | 143 | 68 | 3,377 | 3,588 | ... |
| ... | ... | Brixton, &c. ... ... ... | ... | 10,049 | 609 | 331 | 386 | 55 | 772 | 20 |
| ... | ... | Camberwell Provident ... ... | ... | 53,348 | 939 | 179 | 166 | 562 | 907 | ... |
| ... | ... | Clapham ... ... ... ... | ... | 9,188 | 463 | 152 | 102 | 181 | 435 | ... |
| ... | ... | East Dulwich Provident ... | ... | 15,045 | 981 | 71 | 28 | 874 | 973 | ... |
| ... | ... | Forest Hill Provident ... ... | ... | 14,454 | 484 | 168 | 16 | 290 | 474 | ... |
| ... | ... | Greenwich Provident ... ... | ... | 15,741 | 456 | 41 | 9 | 395 | 445 | ... |
| ... | ... | Wandsworth Common ... ... | ... | 1,666 | 190 | 11 | ... | 179 | 190 | ... |
| ... | ... | Woolwich, &c., Provident ... | ... | 16,453 | 634 | 35 | ... | 600 | 635 | ... |
| 3,614 | 2,836 | | 39,249 | 1,362,549 | 397,185 | 108,275 | 174,857 | 92,630 | 375,762 | 33,656 |

### THE MEDICAL CHARITIES OF LONDON.—A SUMMARY OF THE WORK DONE IN 1916.

It will be seen from the following summary that *One hundred and fifty-nine thousand five hundred and fifty-nine* patients were admitted into the Voluntary Hospitals and Medical Charities of London during the year 1916, and that the attendances in the Out-Patient Departments and Dispensaries numbered *Five millions four hundred and forty-three thousand one hundred and fifty,* at a cost of *£1,729,156.* The Ordinary Income amounted to *£1,556,204,* leaving a deficiency of *£172,952* on the year's work. The Legacies received in 1916 amounted to *£259,145.*

